# Clarification of colloidal and suspended material in water using triethanolamine modified maize tassels

**DOI:** 10.1007/s11356-015-5766-y

**Published:** 2015-11-12

**Authors:** Esther Mbuci Kinyua, Isaac W. Mwangi, Ruth N. Wanjau, J. C. Ngila

**Affiliations:** Department of Chemistry, Kenyatta University, P.O. Box 43844-00100, Nairobi, Kenya; Department of Chemical Technology, University of Johannesburg, Doornfontein Campus, P.O. Box 17011, Doornfontein 2028, Corner Beit and Nind Street, Johannesburg, South Africa

**Keywords:** Flocculant, Turbidity, Quartenized maize tassels, Triethanolamine, Clarification, Nephelometry

## Abstract

Suspended particles in water are a major concern in global pollution management. They affect the appreciation of water due to clarity, photosynthesis, and poor oxygen environment rendering water unsuitable for aquatic animals. Some suspended materials contain functional groups capable of forming complex compounds with metals making them available for poisoning. Such material promotes the growth of bacteria and fouling that give rise to unpleasant taste and odor of the water and thus requires removal. Removal of suspended solids is normally achieved through sedimentation or filtration. However, some suspended colloidal particles are very stable in water and cannot settle while others are able to pass through the filter due to small size, hence difficult to remove. This study investigated the use of triethanolamine-modified maize tassels to form a flocculent for their removal. The modified maize tassels were characterized using Fourier transform infrared (FTIR), and it was found that the triethanolamine was anchored within the cellulose structure of the maize tassels. Clarification parameters such as settling time, reagent dosage, and pH were investigated. The best clarification was at a pH of 6.0 with clearance being less than in 30 min. The optimal flocculent dosage was found to be 3.5 ml of the material, showing that the material has a potential of enhancing clarity in polluted water.

## Introduction

Flocculation is a process of bringing together small particles to form larger particles or settling of colloidal particles from stable suspensions into clump that aggregates into larger particles (Aguilar et al. 2005). The mechanism is based on agglomeration of the very small colloidal particles in the water into flocs, which enable settling, hence clarifying the water (Ebeling et al. 2003). It involves reduction of the charges on the suspended species by addition of suitable chemical compounds (Kawamura 1996). This makes them accumulate into larger particles called flocs which can then settle by gravity and hence easy to remove. Flocculants could be either inorganic such as multivalent metals like aluminum and iron (Sharma et al. 2006) or organic substances. Inorganic flocculants introduce many soluble mineral ions in the water, which have negative health implications (Schintu et al. 2000). As a result, organic flocculants, which are either synthetic or natural water-soluble polymeric materials, are more preferred (Jackson 1981).

Commonly used synthetic polymers for clarification of water include polyacrylamide, polyacrylic acid, polystyrenicsulphonic acid, and polydiallyldimethyl ammonium chloride (DADMAC). However, they have a disadvantage in that they are costly and nonbiodegradable (Lu et al. 2009). There is need therefore for synthesis of flocculants from easily available sources using environmental friendly and sustainable methods for water treatment. Natural polymers, which are mainly polysaccharides, can offer a solution as they have functional groups, which can be modified (derivatized) to produce better flocculants. Such natural products are biodegradable, easily available from renewable resources, and safe for human health. Examples of natural polymers include guar gum, starch, cellulose, and alginic acid among others. Of all the natural polysaccharides, cellulose is the most abundant (Pang et al. 2013).

The hydroxyl groups on the cellulose material can be modified to form a cationic polymer with flocculation properties. This is because it can neutralize or reduce electric charges on the surface of suspended particles promoting collision between the particles and the cation, resulting in cohesion and eventual flocculation as agglomerates (Kleimann et al. 2005). Suspended matter such as algae contains a variety of polar function groups such as sulfated polysaccharide or glycosaminoglycan, which can be agglomerated by the cation due to electrostatic attraction (Love and Percival 1964; Matsubara et al. 2001). Agglomeration, which leads to irreversible bonding, is based on formation of multilayer assemblies of organic systems such as supramolecular functional assemblies or monomolecular layers by a self-assembly process (Knoll et al. 1982; Tillmann et al. 1989). This implies that the cation is capable of removing all suspended matter including finely divided solids from aqueous suspension (Pang et al. 2013).

This study reports the results after the chemical modification of maize tassels using triethanolamine to form a quaternary ammonium compound, which is permanently charged, and its subsequent application on clarification of turbid water. Based on the above, this study has developed a new cation by quaternization of maize tassels. The chemical modification was confirmed by Fourier transform infrared (FTIR) characterization. Flocculation parameters such as settling kinetics, pH, and flocculent dosage were optimized, and the synthesized material was applied for clarification of real water samples.

## Experimental

### Materials and reagents

All the solutions were prepared in double-distilled water, and the reagents were of analytical grade. Triethanolamine, thionyl chloride, pyridine, hydrochloric acid, ammonia, and ethanol were supplied by Sigma-Aldrich (Kobian, Nairobi, Kenya). Maize tassels were obtained from a farm in Gachie sub-county in Kiambu County, Kenya.

### Instrumentation

FTIR spectrophotometer with attenuated total reflectance (ATR) mode (Perkin Elmer 100 with sampling accessory-Waltham, MA, USA)) was used to characterize the parent and the modified maize tassels (Mukamel 2000). The content of suspended matter in both the synthetic water sample and the environmental water samples were determined with the help of a turbidity meter model LP 2000 from HANNA Instruments to determine the extent of turbidity (Wijnen et al. 2014).

### Experimental procedures

#### Quaternization (modification) of maize tassels

A 10 g sample of the biomaterial previously activated at 80 °C for 12 h was suspended in 200 ml pyridine ( in a 500-ml three-necked flask), followed by a dropwise addition of 35 ml of thionyl chloride (SOCl_2_) at 80 °C, under mechanical stirring. After the addition was complete, stirring was continued at the same temperature for another 5 h. The resulting solid was separated by filtration through a Buchner funnel, washed with distilled water, and dried in an oven at 70 °C for 12 h (Tashiro and Shimura 1982). A sample (5 g) of the chlorinated material was placed in a three-necked flask containing 200 ml of ethanol. To this, 100 ml of triethanolamine were added while stirring. The mixture was further mechanically stirred for 7 days and then refluxed at 80 °C for 12 h to obtain the modified liquid material.

### Characterization using FTIR

Analysis of the parent maize tassels, chlorinated, and its modified form was done using FTIR spectrophotometer (FTIR-8400). The FTIR spectra of the parent material, the chlorinated, and the modified form were analyzed, and the functional groups in each were confirmed.

### Settling time

To establish the settling rates, replicate samples (three) were placed in 100-ml measuring cylinders. Different dosages of the flocculant were added to each cylinder, and they were turned agitated to enable thorough mixing of the flocculant reagent with the water sample. The mixture was allowed to settle, and the time taken for the clarification was monitored. This was done by recording the displacement of the settling material from the meniscus of the water at an interval of 10 min for 60 min, until there was no more change in the displacement.

### The jar test

The jar test procedure as demonstrated used by Hudson and Singley (1974) as they determined the effect of pH, dosage on clarification, and the settling rate of both synthetic and real/environmental samples was adopted. The synthetic samples were prepared using known mass of maize flour suspended in water. This was done mixing 1 g of maize flour into 90 ml of distilled water and the mixture was boiled to obtain a uniform suspension. From this, subsequent dilutions were made to obtain the various samples for analysis. A fresh sample was prepared for each analysis.

#### The jar test procedure

Water samples were placed into six beakers and their respective pH adjusted to the desired value using either 0.01 M sodium hydroxide or 0.01 M hydrochloric acid. Different dosages of the flocculant were added to each beaker, and a known volume of the flocculent solution was added. The mixtures were stirred at a constant speed of 100 revolutions per minute (rpm) for 2 min, followed by a slow stirring at 20 rpm for 30 min. The sample was then left to settle for 30 min. Aliquots (10 ml) of the supernatant were drawn (5 cm below the meniscus), and their respective turbidity was measured using a calibrated turbidimeter.

## Results and discussion

The synthesized material was a water-soluble derivative with a pH of 6.0 and had a relative density of 0.85. It was then applied for further experiments, whose results are presented and discussed.

### Characterization and optimization studies

#### FTIR characterization

**Fig. 1:**
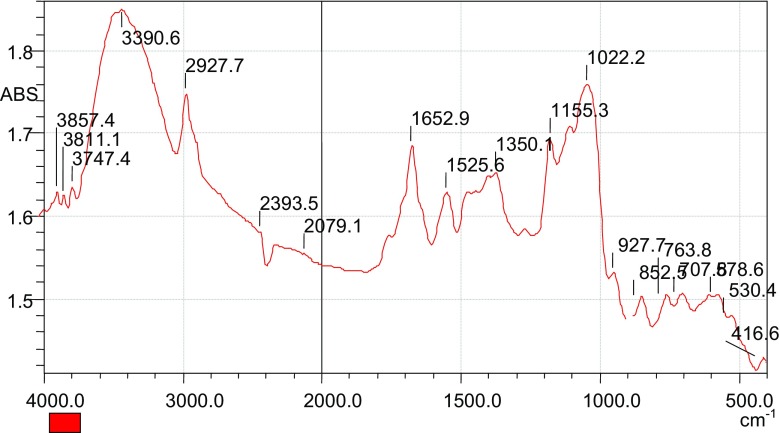
FTIR spectrum of the parent maize tassels

The parent maize tassels were characterized using the FTIR. The results obtained are presented in Fig. 1.

The results from FTIR characterization of the maize tassels indicated the presence of functional groups such as –NH, –OH, and –C=O. The broadband 3390.6 cm^−1^ attributed to either –OH or –NH groups while the band at 1652.6 cm^−1^ could be due to a carbonyl functional group (Nurul et al. 2013; Stuart 1996). Such functional groups could be derivatized to improve flocculation properties of the maize tassels material. The parent material was chlorinated to enable further modification of this product.

#### Characterization of chlorinated maize tassels

**Fig. 2:**
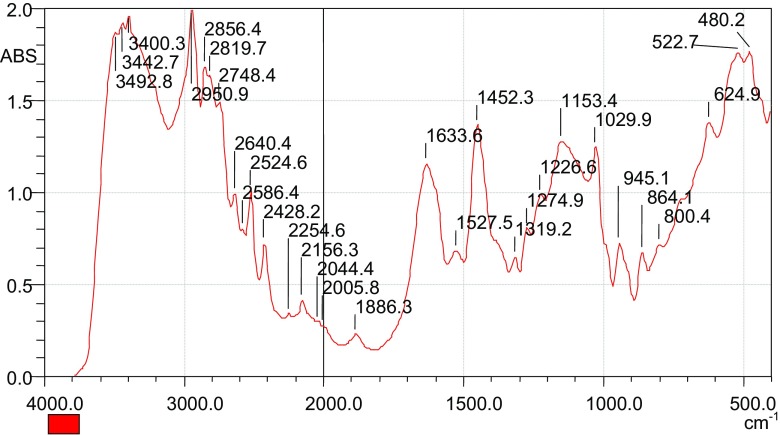
FTIR spectrum of the chlorinated maize tassels

A sample of the chlorinated material was analyzed using the using FTIR, and the results obtained are as presented in Fig. 2.

It was observed that there was a shift of the peaks at 3390.6 to 3400.3 cm^−1^. This could be attributed by the fact that initial peak at 3390.6 cm^−1^ could have been contributed by the –OH group which changes upon chlorination. A new peak at 624.9 cm^−1^ appears, indicating the presence of the –C–Cl stretch. This peak was absent in the parent maize tassels. This implies that a chloro group was introduced into the maize tassels. The chloro group was then condensed with a tertiary amine.

#### Quartenization of the chlorinated maize tassels

**Fig. 3:**
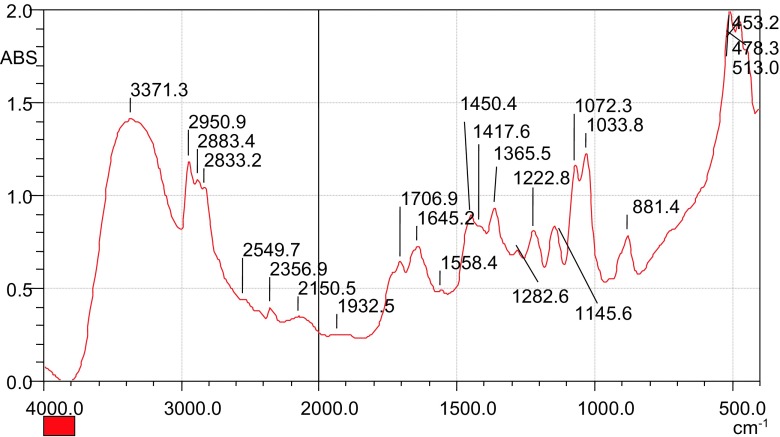
FTIR spectrum of the quartenized maize tassels (QMT)

The chlorinated maize tassels were reacted with triethanolamine for the quaternization process of the chlorocellulose to occur. The resulting FTIR spectrum obtained is as presented in Fig. 3.

It was observed that the peak at 624.9 cm^−1^ disappeared showing that the –C–Cl group had disappeared after the chloro group being replaced by an amine group. A shift of the peak from 3400.3 to 3371.3 cm^−1^ was also observed, which suggests the presence of an amine group, –NH_4_^+^ or its derivates, and it is also shown by the appearance of a peak at 2356 cm^−1^. A new peak at 1365.5 cm^−1^ can be attributed to the presence of the amide functional group, which is a characteristic of charged –NH_4_^+^ functional groups (Kacurakova et al. 2006). The peak at 1645.2 cm^−1^ was due to the –NH group (Carden and Morris 2000). The above results show that there was the presence of cationic sites, which can interact with colloidal particles in water for effective clarification (Weber 1986). This confirms that the modification was successful, and the material was applied for flocculation experiments.

### Clarification parameters

#### Settling time

**Fig. 4:**
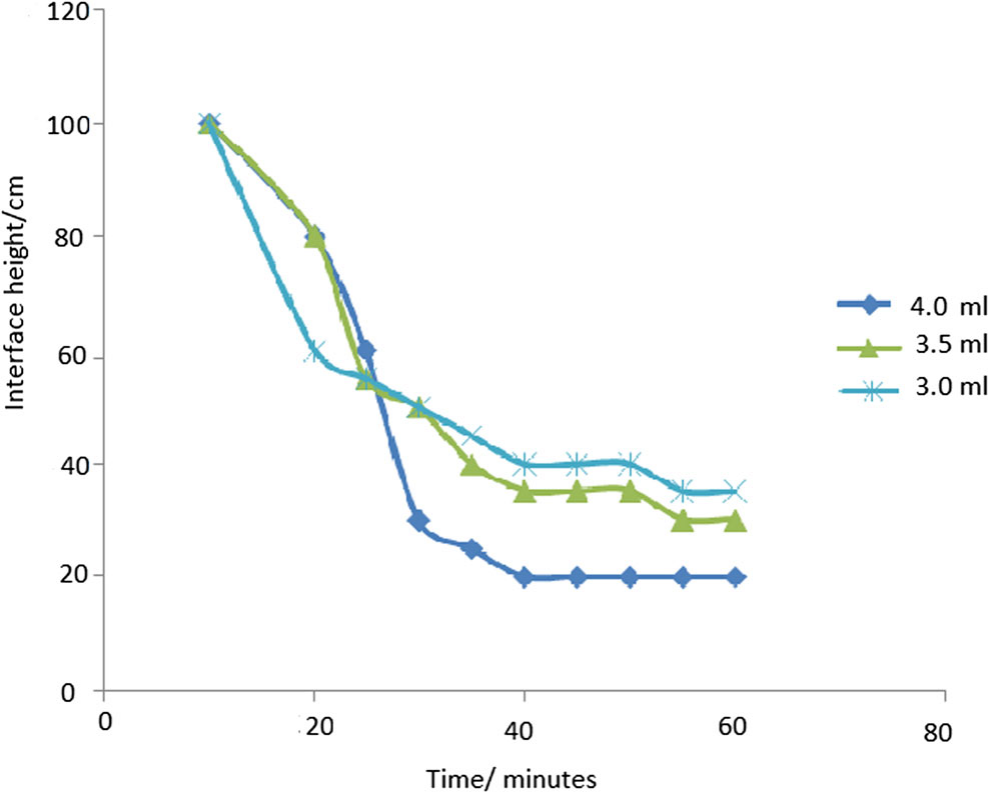
Settling of suspended material at pH 6 with different QMT dosages

Kinetics of clarification describes the rate of electrostatic interaction between the polyelectrolyte on the surface of the colloidal material and which governs the formation of multilayer assemblies (Barker et al. 2000). This contributes to the formation of flocs which result to agglomeration. The agglomerated material settles to the bottom due to attraction by gravitational force. As the material settles, the liquid starts to clear from the top. The effect of settling time on the clarification of synthetic water samples was monitored by recording the displacement of the settling material from the meniscus of the water in the jar. It was carried out using synthetic samples buffered at varied pH vales of 4.0, 6.0, 6.5, 7.0, and 8.0. It was found out that pH 6.0 yielded the best results. This could be due to the fact that some quaternary polycations lose their cationic nature at pH higher than 6.5 (Badawy and Rabea 2001). At lower pH values, the presence of positively charged protons offers a competition to the colloidal material. Further clarification experiments were done at a pH value of 6.0. To establish the settling rates, three samples containing 100 ml of the synthetic water (pH 6.0) of turbidity (53.8 NTU) were treated with three different quaternized maize tassels (QMT) reagents (3.0, 3.5, and 4.0 ml), and the results obtained are presented in Fig. 4.

**Fig. 5:**
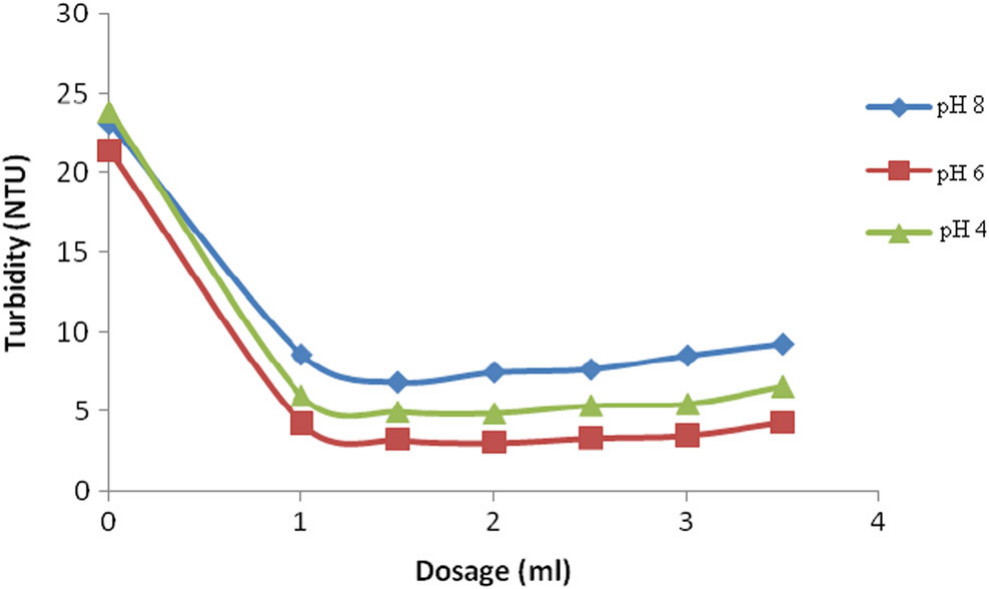
Jar test results of reduced turbidity using QMT at different pH values

The results showed that the rate of settlement of suspended particles in the synthetic turbid waters was quick such that the suspended matter settled within the first 30 min. It was also observed that the rate of settling was proportional to dosage of the triethanolamine-modified maize tassels. This is because at a high dosage of the QMT (4.0 ml), there was a high number of the polycations present in the water. These polyelectrolytes attached onto the colloids due to electrostatic attraction, and since the polymeric chain is long, the mobility of the colloid-polymer particle is low. This promoted contact with other particles as the polymer chain protruded to form “bridges” with other similar particles. Therefore, there was particle agglomeration into flocs, which settled down due to their high density. The study observed that 30 min were sufficient to carry out flocculation experiments (Fig. 5).

#### Effect of pH on clarity

The effect of pH on the rate of clarification was monitored. This is because pH influences the charge of nitrogen-containing organic compounds (Kairi and Kassim 2013). The charge density of amino groups is influenced by pH and affects their dissociation constants (pKa), and thus the interaction of the polycation and the suspended material (Sorlier et al. 2001).

The jar test procedure was used with the test samples (250 ml) having a turbidity of 24 NTU and buffered at different pH environments of 4.0, 6.0, and 8.0. The turbidity of the supernatant samples drawn out of each jar and the clarity was measured after 30 min using a calibrated turbidimeter.

The results show a clarification profile of general increase in clarity for all the three samples. However, pH 6.0 provided the best clarification as it had the lowest turbidity as compared to the rest. This can be attributed to the high flocculant activity of the polyelectrolyte because quaternary amine polyelectrolytes become highly cationic at that pH value. At this pH (6.0), there was increased neutralization of the particles by the flocculant and high bridging effect due to the spreading of its molecules (Xing et al. 2013). At pH 4.0, the environment has a high positive charge contributed by the protons of the acidic media offering a completion to attract negatively and neutral species in water (Sahu and Chaudhari 2013). The presence of H^+^ ions in solution lowered the negative surface charge of the particles because of the acidic nature of the solution, resulting to particles tending to self-aggregate (Sahu and Chaudhari 2013). At pH 8.0, the polyelectrolyte becomes negatively charged and this restricts agglomeration to neutral species in the water only due to static electrical attraction (Shin et al. 2006). The polyelectrolyte was effective in lowering water turbidity contributed by neutral species at pH 8.0. The best flocculation results were at pH 6.0, which is at the physiological pH of water, and further flocculation experiments were carried out at that pH value.

#### Effect of dosage on clarity

**Fig. 6:**
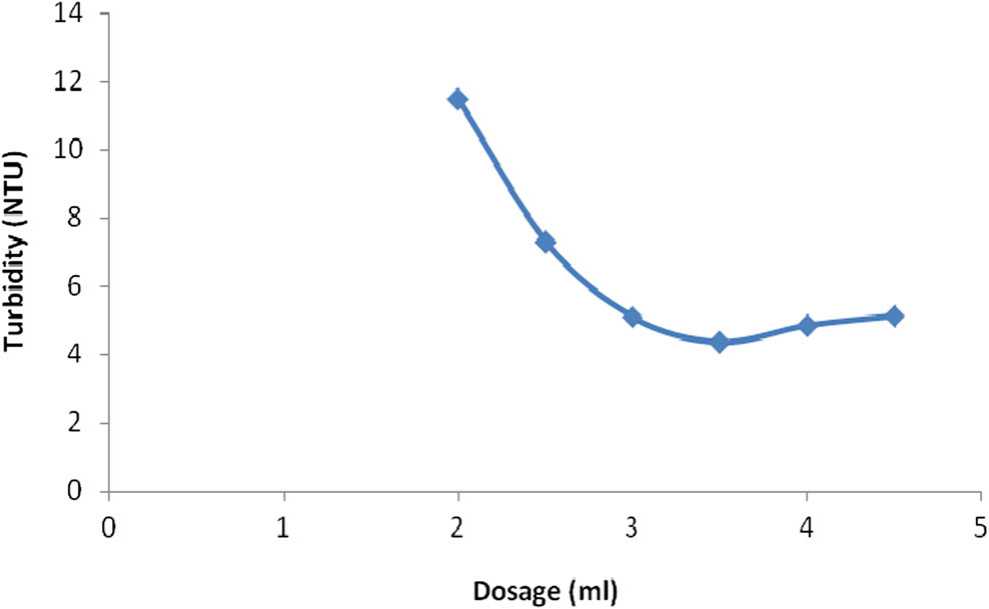
Effect of flocculent dosage on clarity of water

The effect of reagent dose was investigated using water with a highly suspended mater. The jar test procedure was carried on waters having a turbidity of 12 NTU while varying dosages of the QMT. The turbidity of the supernatant samples drawn after 30 min and measured using a calibrated turbidimeter. The turbidity values obtained were plotted against the concentration as shown in Fig. 6.

The results show a general decrease in turbidity with increase in the dosage of the QMT reagent. The lowest turbidity of 4.36 NTU was achieved at a dosage of 3.5 ml of the synthesized clarifying reagent at the pH of 6.0. This is because as the QMT encounters a colloidal particle, its positive sites are attracted to the particle surface reducing the repulsive forces between the colloidal particles, the remainder of the molecule remain extended into the solution forming bridges with other similar particles. This causes agglomeration of the colloidal particles into flocs, which then settle by gravity due to increased density of the resultant particle. When the flocculating reagent has completely neutralized the anionic charges of the particles, the turbidity level of the synthetic water reaches its minimum and this is the optimal QMT dosage. Further increase in the clarifying reagent had no effect on the change in the turbidity of this synthetic water sample. The negative charges on the agglomerated colloidal particles cause repulsion between themselves as they collide with each other and prevent Van der Waals interactions contributing to the observation (Cayre et al. 2003). Therefore, the clarifying dosage at 3.5 ml was the most effective dosage of the flocculant when the turbidity of water was 12 NTU.

#### Effect of high turbidity on clarification

**Fig. 7:**
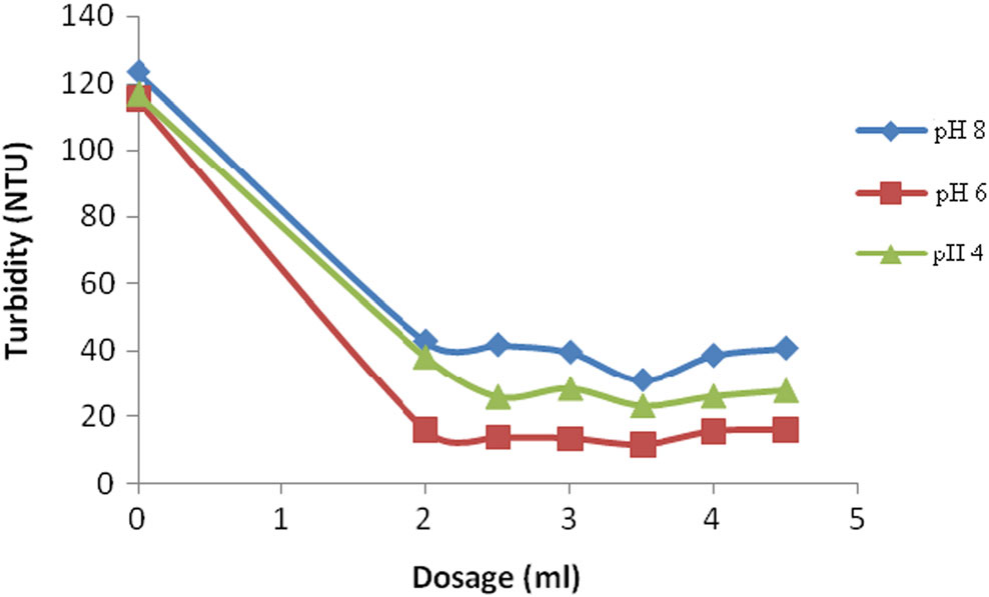
Effect of QMT dosage at different pH values on high turbid waters

Other experiments were carried using high turbid water samples (50 to 150 NTU) while the synthetic water was buffered at different pH values. The results obtained are provided in Fig. 7.

It was observed that good clarification was achieved at pH value of 6.0 which favors the presence of lower positively or even negatively charged species when the turbidity was low. At that pH, the material had a high potential as it had its cationic nature (Badawy and Rabea 2001). This suggests that even at high turbidity, flocculation is due to heterocoagulation caused by charge neutralization of supermolecular layers that provide an effective aggregation of the particles since the charges of the particle are not neutralized (Lim-Seok 1994). The study therefore observed that the synthesized material was an effective flocculent at pH 6.0, hence suitable for use on environmental water samples at varying dosages of the flocculant. This demonstrates that concentration of the interacting species enhances the flocculation process as they interact in certain stoichiometric ratios (Knoll et al. 1982; Tillmann et al. 1989). If the dosage of the QMT was increased, the clarity of turbid water could be made to suitable levels. The results show that as the dosage increases, the clarity of water increases to a value of 20 NTU after which no more clarification was observed. This is attributed to the fact that suspended particles are destabilized by the electrostatic repulsion among themselves even when they are already bound with the quaternary maize tassels resulting into increase in turbidity as reported by Weber (1986).

### Application of the QMT on environmental water samples

**Fig. 8:**
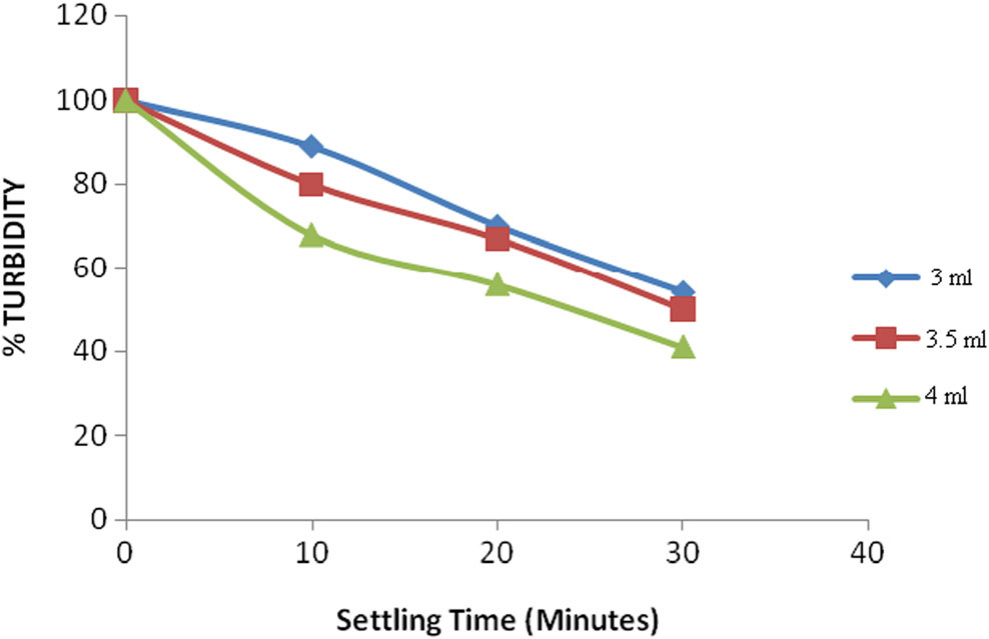
Jar test results on application of the optimum conditions on environmental water sample

The environmental water samples were collected from a swampy field in Wangige, Kiambu County, in Kenya. The water sample had an initial turbidity of 25.14 NTU at a temperature of 24.0 °C and a pH of 7.0. The samples were treated with 3.0, 3.5, and 4.0 ml of the QMT dosage using the jar test procedure, and the turbidity of their supernatant samples was drawn and measured at intervals of 10 min during settling time. The results obtained are shown in Fig. 8.

These results are in agreement with the results obtained using the synthetic water; that concentration of the QMT is the driving force in the clarification process.

### Analysis of the sludge

**Fig. 9:**
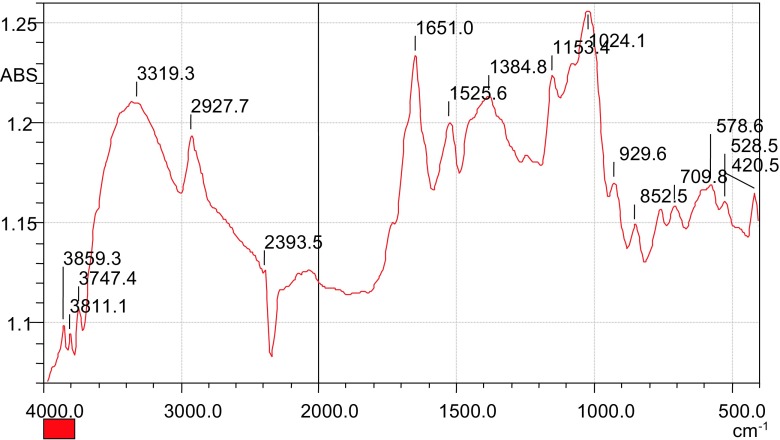
FTIR spectrum of the settled sludge after clarification

The clarification process results into formation of a floc which is herewith referred to as sludge. A sample of the sludge was analyzed by FTIR, and the resulting spectrum is shown in Fig. 9.

**Fig. 10:**
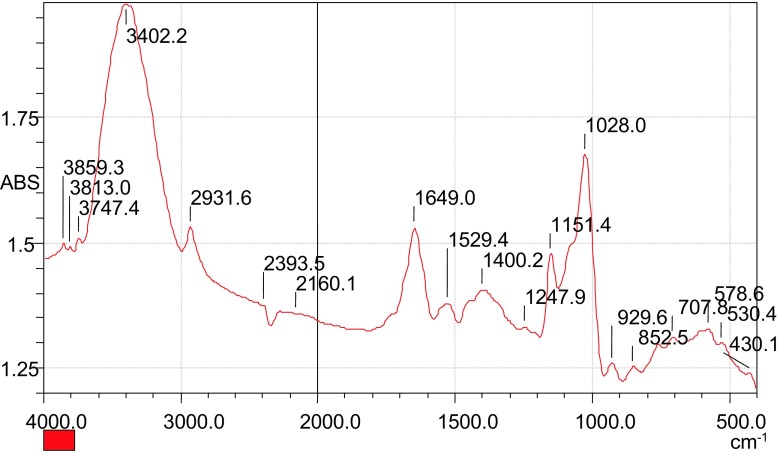
FTIR spectrum of the sludge after treating it with hydrochloric acid

The results showed presence of free O–H groups at the peaks 3859 and 3811 cm^−1^. The peak at 1384.8 cm^−1^ was attributed to the presence of NH_4_^+^. This shows that the charged species was still in the sludge, confirming that the QMT was co-precipitated with the settling flocs during clarification process. This may be due to formation of an irreversible adsorption of a polyelectrolyte layer on the surface of those suspended particles forming a strong bond (Barker et al. 2000). The bond is based on the formation of layer-by-layer assemblies leading to the formation of supermolecular layers by a self-assembly process (Knoll et al. 1982; Tillmann et al. 1989). This implies that the polycation settles with the agglomerated matter as sludge. The resulting sludge formed during flocculation process was treated with hydrochloric acid and the results obtained are presented in Fig. 10.

It was observed that the peak at 1365.5 cm^−1^ shifted to 1400.2 cm^−1^. The shift could be due to the attachment of the chloride ion on the charged NH_4_^+^ group. This implies that the polycation was strongly attached to the sediments due to the strong bond of the layer-by-layer assemblies suggested by Knoll and co-workers (1982).

## Conclusions

The study successfully synthesized a quaternary ammonium compound by modifying powdered maize tassels with triethanolamine, which was confirmed by FTIR analysis. The polycationic material was observed to have high flocculation properties such that a sample whose turbidity was 53.8 NTU was settled in less than 30 min. Its flocculation capacity was such that water with a turbidity of 12 NTU required 3.5 ml to bring it to a clarity level of 4.36 NTU in less than 30 min. This value is within the recommended level by the WHO (Pichler et al. 2012).

The polyelectrolyte was a positively charged cation that interacted with suspended matter in water and strongly attached to each other and cannot easily be detached. The co-precipitation process interacted on stoichiometric ratios such that the concentration was the driving force in settling of the colloidal and suspended matter, and could be depleted from the solution and therefore a suitable method for clarification. The clarification process was found to be best at a pH value of 6.0, which is close to the physiological pH of water. This study offers a solution to addressing the problem of water pollution by suspended and colloidal matter through treatment with triethanolamine-modified maize tassels.
